# Presence of Brain Metastases at Initial Diagnosis of Cancer: Patient Characteristics and Outcome

**DOI:** 10.7759/cureus.4113

**Published:** 2019-02-21

**Authors:** Carsten Nieder, Ellinor Haukland, Bård Mannsåker, Adam R Pawinski, Rosalba Yobuta, Astrid Dalhaug

**Affiliations:** 1 Oncology, Nordland Hospital Trust, Bodø, NOR

**Keywords:** brain metastases, prognostic factors, radiotherapy, surgery, synchronous metastases

## Abstract

Objective

To describe the characteristics of patients who present with brain metastases already at first diagnosis of cancer and to evaluate overall survival (OS) and long-term survival.

Methods

Retrospective uni- and multivariate analyses in a group of 84 patients treated with different approaches.

Results

With respect to primary cancer type, the largest entities were adenocarcinoma non-small cell lung cancer (NSCLC) and small cell lung cancer (SCLC) (34.5 and 21.4%, respectively). The most common diagnostic setting was symptomatic brain metastases (64 patients, 76.2%). Median OS was 7.2 months (one-year survival rate 31%). Four patients survived for at least three years, all had solitary metastases. The best survival was observed in the group managed with neurosurgical resection, median 17.7 months. Systemic treatment was also associated with better survival (median 9.7 vs. 2.8 months, p = 0.0001). Multivariate analysis revealed two prognostic baseline factors for OS, Karnofsky performance status (KPS) and number of brain metastases. Neurologic cause of death was uncommon (n = 14, 17%).

Conclusion

Long-term survival was limited and observed exclusively in the setting of a solitary brain metastasis. In patients with good KPS and limited number of brain metastases, systemic treatment as well as effective local treatment, such as resection and/or radiotherapy with sufficiently high equivalent dose, is warranted.

## Introduction

The setting in which brain metastases are diagnosed is very heterogeneous and includes radiological screening to determine the eligibility for certain treatment approaches, and also clinical symptoms in patients already diagnosed with cancer, among others [1]. Occasionally, neurological and/or cognitive deficits are the first clinical sign of an intracranial tumor, and some of these lesions turn out to represent distant seeding from extracranial primary tumors [2]. Regardless of diagnostic setting, brain metastases impact on patients' prognosis and healthcare resource utilization [3]. Number, size and location of newly diagnosed brain metastases are highly variable, also in patients who present with such lesions when they are diagnosed with cancer for the first time. The different scenarios even include solitary brain metastases in patients with early-stage local disease, e.g., in the lung [4]. Using data from 18 SEER registries (the National Cancer Institute's Surveillance, Epidemiology, and End Results system) from 2010 to 2013, Kromer et al. assessed the frequency of brain metastases at the time of primary diagnosis in the US [5]. There were 1,634,954 total primary cancer cases in SEER from 2010 to 2013, 1.7% of which presented with synchronous brain metastases. The cancer type with the highest proportion was lung cancer (10.8% of cases with initial brain metastases), followed by esophageal (1.5%), kidney (1.4%), and melanoma (1.2%). In a different study performed in Japan by Nozawa et al., only 0.1% of patients with colorectal cancer had brain metastases at initial diagnosis [6]. Because relatively few researchers have reported on baseline features and prognosis of patients with synchronous brain metastases at first cancer diagnosis, we retrospectively analyzed our institution's database. We were particularly interested in the likelihood of long-term survival in this setting.

## Materials and methods

Our institution has previously established an electronic database for retrospective quality of care analyses, which has collected baseline, treatment and outcome data of all patients with parenchymal brain metastases from solid primary tumors managed since 2007 [7, 8]. For the present study, all patients seen between 2007 and end of 2016 were extracted. Of these, 74 were excluded because they did not receive any active oncological treatment. Among the remaining 332 patients, 84 (25%) were diagnosed with synchronous brain metastases at the time of initial cancer diagnosis. These 84 patients were included in further statistical analyses.

Treatment was highly individualized and included surgery, local and/or whole-brain radiotherapy (WBRT) and, if necessary, salvage with repeat surgery and/or radiotherapy. The choice between different WBRT fractionation regimens was at the discretion of the radiation oncologist. Often, 10 fractions of 3 Gy were prescribed. Patients with adverse prognostic features were also treated with five fractions of 4 Gy. Sequential systemic therapy was at the discretion of the medical oncologists. If deemed appropriate by the multidisciplinary tumor board, patients with lung cancer and asymptomatic, imaging-detected brain metastases started systemic therapy first, usually four cycles of platinum-based doublet chemotherapy. Afterwards WBRT or stereotactic radiosurgery (SRS) was employed. Patients with small cell lung cancer (SCLC) always received WBRT as their first local treatment, with SRS reserved for subsequent salvage. Local treatment of the primary tumor (T) and nodal (N) sites was also discussed by the hospital’s multidisciplinary tumor boards. Strategies included curative surgery, radiochemotherapy, radiotherapy alone and systemic treatment only. Actuarial survival from day of first treatment was calculated with the Kaplan-Meier method and compared between different groups with the log-rank test. Seven patients were alive at last recorded follow-up and censored in the actuarial survival analyses. Date of death was entered in all other patients. The median follow-up was 27 months (range: 1.5–78 months) in censored patients. Relevant prognostic factors for overall survival, defined as log-rank test with p < 0.1, were entered in a multivariate forward stepwise conditional Cox model. IBM SPSS 24 (IBM, Armonk, NY) was employed for these analyses.

Identical to our previous retrospective analyses [7, 8], no approval from the Regional Committee for Medical and Health Research Ethics (REK) was necessary. Similarly no approval from the Norwegian Social Science Database (NSD) had to be obtained.

## Results

The median age was 66 years (range: 41–90 years). With respect to primary cancer type, the largest groups were patients with adenocarcinoma non-small cell lung cancer (NSCLC) and SCLC (34.5 and 21.4%, respectively). Table 1 shows the distribution of histologies.

**Table 1:** Primary cancer type, n = 84. NSCLC: Non-small cell lung cancer; SCLC: Small cell lung cancer; na: Not applicable.

Tumor type	Number	Percent	Median overall survival in months
NSCLC, adeno carcinoma	29	34.5	8.4
NSCLC, squamous cell carcinoma	11	13.4	4.7
NSCLC, other	6	7.1	5.9
SCLC	18	21.4	9.7
Renal cell cancer	8	9.5	7.1
Malignant melanoma	5	6.0	12.7
Breast cancer	2	2.4	na
Colon cancer	2	2.4	na
No primary found	2	2.4	na
Small bowel cancer	1	1.2	na

The most common diagnostic setting was symptomatic brain metastases (64 patients, 76.2%). Among 20 patients with asymptomatic imaging-detected lesions, two had malignant melanoma, one renal cell cancer, and the others lung cancer. Diameter of the index lesion was significantly larger in symptomatic patients (median 2.55 vs. 1.0 cm, mean 2.75 vs. 1.0 cm, standard deviation 1.3 and 0.5 cm, respectively), p = 0.0001.

**Figure 1:**
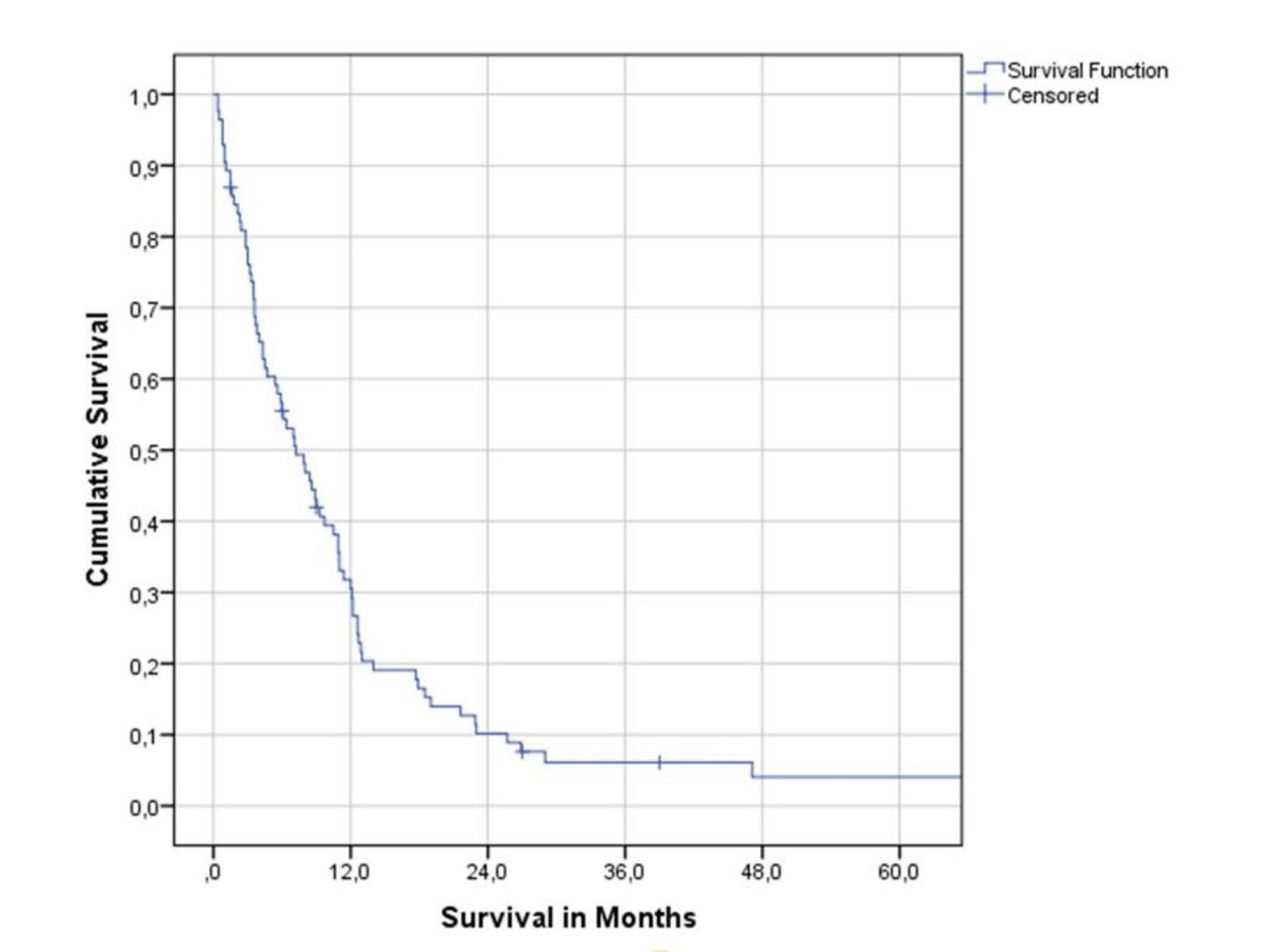
Overall survival (Kaplan-Meier estimate).

Median overall survival was 7.2 months (one-year survival rate 31%, two-year rate 10%), as shown in Figure 1.

Four patients survived for at least three years, including one with SCLC (solitary brain metastasis, WBRT, curative thoracic radiochemotherapy), one with renal cell cancer (solitary brain metastasis, SRS, nephrectomy), and two with squamous NSCLC (solitary brain metastasis, surgery, chemotherapy, palliative thoracic radiotherapy) (solitary brain metastasis, surgery, curative thoracic radiochemotherapy). Neurologic cause of death was uncommon (n = 14, 17%), most patients died from extracranial tumor progression.

Table 2 shows the different upfront treatment strategies.

**Table 2:** Upfront treatment strategies, n = 84. SCLC: Small cell lung cancer; WBRT: Whole-brain radiotherapy; SRS: Stereotactic radiosurgery; FSRT: Fractionated stereotactic radiotherapy.

Treatment	Number	Percent	Median overall survival in months
Chemotherapy, SCLC	16	19.1	9.7
Chemotherapy, other	4	4.8	2.4
Surgery with or without WBRT	12	14.3	17.7
SRS or FSRT	8	9.5	9.0
WBRT	44	52.4	3.8

The best survival was observed in the group managed with neurosurgical resection, median 17.7 months (p = 0.004, pooled over all strata). Among 18 patients with SCLC, 16 started their treatment with systemic chemotherapy, while two received WBRT before the first cycle of chemotherapy. Table 3 shows the median survival after different radiotherapy regimens, also taking into account subsequent salvage.

**Table 3:** Radiotherapy regimens, n = 84. WBRT: Whole-brain radiotherapy; SRS: Stereotactic radiosurgery; FSRT: Fractionated stereotactic radiotherapy. *Two patients who should have received chemotherapy followed by WBRT died during chemotherapy. **Two patients did not receive all 10 fractions due to rapid clinical deterioration.

Treatment	Number	Percent	Median overall survival in months
No radiotherapy at all	5*	6.0	3.6
Any SRS or FSRT	10	11.9	11.0
WBRT + boost	8	9.5	7.1
WBRT 30 Gy in 10 fractions	55**	65.5	6.4
WBRT 20 Gy in five fractions	6	7.1	5.6

Overall, 11 patients (13.1%) received any local salvage therapy (resection, SRS, other radiotherapy). Compared to WBRT-based regimens, those including SRS or fractionated stereotactic radiotherapy (FSRT) were associated with significantly longer survival (p = 0.04, pooled over all strata). Overall, 69 patients (82.1%) received WBRT at some point in time. Systemic treatment was also associated with better survival (median 9.7 vs. 2.8 months, p = 0.0001).

Regarding prognostic factors for overall survival, Table 4 shows the results of uni- and multivariate analyses. The latter revealed that Karnofsky performance status (KPS) and number of brain metastases were independently associated with this endpoint.

**Table 4:** Prognostic factors for overall survival, n = 84 (log-rank test, multivariate Cox regression analysis). KPS: Karnofsky performance status *For example bone(s), example for more than one: liver and lung(s)

Parameter	Number	Percent	Median overall survival in months	Univariate p-value	Multivariate p-value
Female gender	40	47.6	6.0		
Male gender	44	52.4	8.4	0.59	Not included
Symptomatic metastases	64	76.2	6.1		
Imaging detected metastases	20	23.8	10.5	0.60	Not included
No extracranial metastases	30	35.7	11.0		
One extracranial organ*	29	34.5	8.4		
More than one extracranial organ	25	29.8	3.7	0.002	0.08
KPS 40-50	7	8.3	3.0		
KPS 60	9	10.7	6.0		
KPS 70	26	31.0	4.5		
KPS 80	17	20.2	7.9		
KPS 90-100	25	29.8	17.7	0.0001	0.0001
Age <=65 years	40	47.6	11.0		
Age >=66 years	44	52.4	5.9	0.06	0.08
Single brain met.	25	29.8	11.4		
Two brain met.	13	15.5	7.9		
Three brain met.	16	19.1	5.6		
4-9 brain met.	21	25.0	4.3		
10 or more brain met.	9	10.7	3.0	0.02	0.05

## Discussion

This study of 84 patients with synchronous brain metastases (25% of all actively treated patients in the database) confirmed literature data, which suggested that metachronous presentation is more common [2, 5, 6]. Our study included a large proportion of patients with lung cancer and is also characterized by frequent use of WBRT. Most patients had multiple brain metastases and also extracranial metastases. Survival was better in patients who received systemic therapy and more efficacious local brain-directed approaches, such as resection and SRS. However, selection bias may have contributed to these differences. As in a recent study by Shibahara et al., symptomatic lesions were more common than asymptomatic lesions [9]. Of 471 patients with brain metastases in their study, 93 (20%) were included in the synchronous group (25% in our study), 76 (16%) in the group with short interval of maximum two months, and 302 (64%) in the metachronous group. There were no differences in OS from the detection of brain metastases among the three groups in univariate and multivariate analyses. A study by Choi et al., limited to renal cell cancer, found no differences between synchronous and metachronous presentation in terms of lesion progression and OS after the diagnosis of brain metastases [10].

In our lung-cancer-dominated study, median OS was approximately seven months and very few patients (5%) experienced long-term survival of three or more years. In contrast, a SEER study limited to synchronous brain metastases from breast cancer showed that 21% of the patients were alive at three years (median OS 10 months) [11]. There were substantial differences in OS according to tumor subtype, with triple-negative disease having the worst outcome. The number of patients with breast cancer was not sufficient for subgroup analyses in our study. Ho et al. collected information on 992 breast cancer patients with brain metastases and/or leptomeningeal disease, whose primary tumor was diagnosed between 2004 and 2010 [12]. Of these, 165 patients had synchronous metastases (16.6%). Median OS was 5.0 months (similar for synchronous and metachronous presentation). Non-triple-negative breast cancer and systemic therapy were associated with improved OS in both groups. In patients with synchronous metastases, surgery for the primary tumor and the metastases also improved survival.

Regarding the common scenario of NSCLC with synchronous brain metastases, data analyzed by Lind et al. (n = 167) showed a median OS of 12.1 months if the patients underwent neurosurgery/SRS [13]. Median OS of WBRT patients was 3.7 months. Those undergoing radical thoracic treatment (n = 24) had a longer median OS (28.4 months) than those undergoing chemotherapy (n = 74; 12.1 months) or supportive therapy (n = 69; 5.6 months, p < 0.01). Patients with stage I thoracic disease (n = 23) had a longer median OS (18.5 months) than those with stage III (n = 43; 9.4 months). Performance status and age were also associated with OS.

A different study retrospectively analyzed NSCLC patients with 1-4 synchronous brain-only metastases and excluded those with KPS < 70 [14]. Aggressive thoracic therapy was defined as resection of the primary disease or radiochemotherapy whose total radiation dose exceeded 45 Gy. Sixty-six patients were included. Intrathoracic disease extent included nine stage I, 10 stage II and 47 stage III patients. Thirty-eight patients received aggressive thoracic therapy, and the latter was associated with prolonged median OS (26.4 vs. 10.5 months; p < 0.001). In NSCLC patients with synchronous brain metastases treated with lung surgery, five-year survival rates of approximately 20–25% have been reported [15, 16]. Taken together, several retrospective studies suggest that aggressive management of thoracic disease may be associated with improved OS.

Regarding prognostic factors, one study recommended use of the lung cancer-specific graded prognostic assessment (GPA), which includes KPS, number of brain metastases, extracranial metastases and age [17]. However, only patients with 1-3 brain metastases were included. A different group developed a prognostic model specifically for NSCLC patients with brain metastases at the initial diagnosis [18]. The model was derived using data from 1158 consecutive patients, with 837 in the derivation cohort and 321 in the validation cohort. These authors established two prognostic models for the whole group of patients and for those with known epidermal growth factor receptor (EGFR) genotype, respectively. Six factors were independently associated with survival time: KPS, age, smoking history (replaced by EGFR mutation in model 2), local treatment of intracranial metastases, EGFR-tyrosine kinase inhibitor treatment, and chemotherapy. Patients were stratified into low- (score, 0-2), moderate- (score, 3-5), and high-risk (score 6-7) groups according to the median survival time (16.6, 10.3, and 5.2 months, respectively; p < 0.001). This approach is difficult to compare to other models, because treatment-related variables were factored in. Moreover, given the lack of significant prognostic implications of the diagnostic setting (synchronous vs. metachronous) [9], separate models for patients with metachronous metastases are of uncertain clinical value. Our own patient cohort could not be assessed according to this model, because smoking history was not recorded. Furthermore, EGFR mutations are found in less than 5% of the patients in our geographical region.

Previous studies have identified subgroups of patients with very unfavorable prognosis, e.g., median OS of two months or less, which might be appropriate for best supportive care without brain-directed treatment [7, 19]. As shown in Tables 3, 4, none of the subgroups in the present study fell into this category. While careful judgment is warranted regardless of prognosis, our results suggest that patients with 10 or more brain metastases or KPS <60, who had median OS of 3.0 months, may be at relatively higher risk of overtreatment. Due to the limited size and statistical power of our study, further analyses of patients with synchronous brain metastases are recommended.

## Conclusions

Long-term survival was limited and observed exclusively in the setting of a solitary brain metastasis. In patients with good KPS and limited number of brain metastases, systemic treatment as well as effective local treatment, such as resection and/or radiotherapy with sufficiently high equivalent dose, is warranted. Given that neurologic death was uncommon, improvement of extracranial disease control is of high importance.
